# Inflammatory mediators of proliferative vitreoretinopathy: hypothesis and review

**DOI:** 10.1007/s10792-020-01325-4

**Published:** 2020-02-26

**Authors:** Ying Dai, Chenghua Dai, Tao Sun

**Affiliations:** 1 Department of Ophthalmology, The First People’s Hospital of Yancheng, No. 10, Nancheng River Road, Yancheng, 224000 Jiangsu Province China; 2 Department of Ophthalmology, Yangzhou Hospital of Traditional Chinese Medicine Affiliated to Nanjing University of Chinese Medicine, Yangzhou, 225000 Jiangsu Province China

**Keywords:** Proliferative vitreoretinopathy (PVR), Inflammatory mediators, Growth factors, Cytokines, Treatment

## Abstract

**Purpose:**

To review the role of inflammatory mediators in proliferative vitreoretinopathy (PVR) development and the current treatment for PVR prevention.

**Methods:**

A PubMed search was carried out using these keywords “PVR,” “inflammatory mediators,” “growth factors,” “cytokines” and “treatment.” Studies regarding inflammatory mediators and PVR therapy were included and published up to December 2019.

**Results:**

Inflammatory mediators, namely growth factors and cytokines, have been implicated in the occurrence and development of PVR. Among various inflammatory mediators, transforming growth factor-β, platelet-derived growth factor, interleukin-6, interleukin-8 and tumor necrosis factor-α are considered to be particularly important. In this review, we focus on the hypothesis that growth factors and cytokines are involved in the development of PVR, and current treatment for the prevention of PVR.

**Conclusion:**

We support the hypothesis that growth factors and cytokines may participate in the complex process of PVR development. More importantly, the identification of inflammatory mediators provides novel and efficacious therapeutic targets for the treatment of PVR.

## Introduction

Proliferative vitreoretinopathy (PVR) was coined by the Retina Society Terminology Committee in 1983 to describe a disease process occurring secondary to rhegmatogenous retinal detachment (RRD) [1–3]. PVR is estimated to cause approximately 5–10% of all retinal detachments [4, 5]. Patients with a long history of untreated RRD, large retinal breaks or retinal tears, multiple retinal breaks, extensive detachments or choroidal detachment are at a higher risk of progressing into PVR [4, 6]. In addition, PVR can occur in patients with ocular trauma, intraocular inflammation or after retinal procedures including retinal cryopexy, laser retinopexy, pneumatic retinopexy, scleral buckle or pars plana vitrectomy (PPV) [4, 7, 8]. Currently, surgery is the standard PVR treatment [9]. However, as the major postoperative complication of RRD, PVR is responsible for the failure of retinal reattachment surgery [2, 10, 11]. Therefore, more attention has recently been paid to the pathogenesis and treatment of PVR. To date, the pathogenesis of PVR is not yet fully understood.

**Fig. 1:**
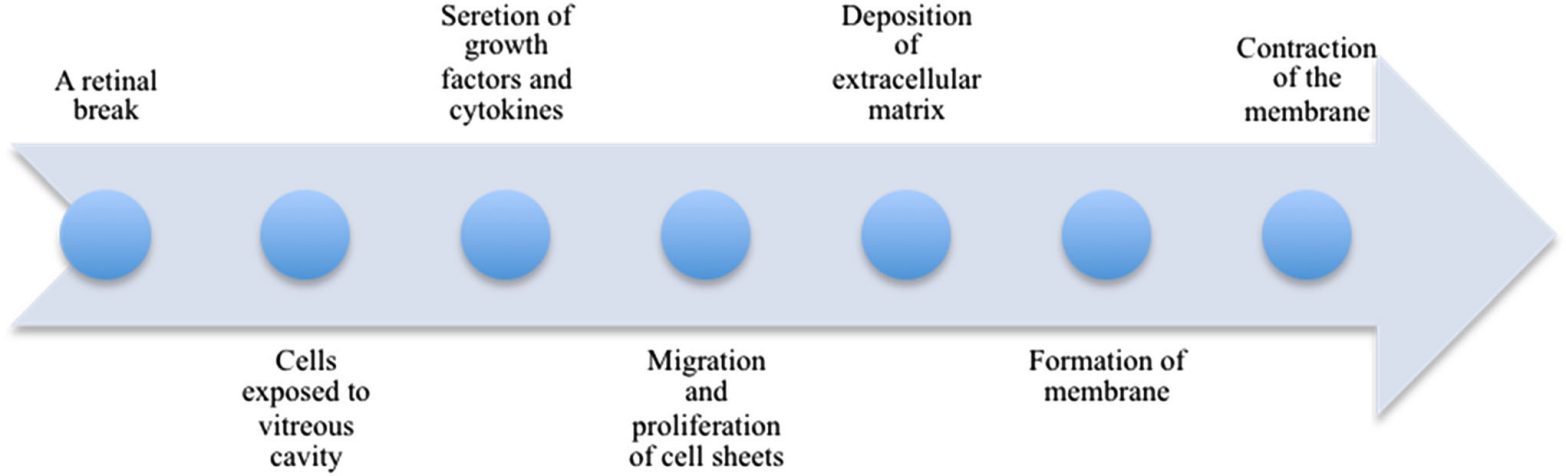
Growth factor and cytokine hypothesis for the development of PVR. Following a retinal break, RPE cells are exposed to vitreous cavity to contact with growth factors and cytokines, resulting in more growth factors and cytokines secretion and cellular responses stimulation (such as cellular migration and proliferation as well as deposition of extracellular matrix). As a result, the epiretinal membrane formation and contraction cause retina re-detachment and PVR development. Growth factors and cytokines are tightly associated with these complex processes

Following a retinal break, the retinal pigment epithelial (RPE) cells are exposed to vitreous cavity to react to growth factors and cytokines in the vitreous, resulting in a forward feedback to secret more growth factors and cytokines to further stimulate cellular responses [4, 12]. Fibrous astrocytes, fibroblasts, myofibroblasts and macrophages were also involved in the development of PVR [9, 13]. In eyes with PVR, RPE cells and myofibroblasts are the predominant cell type [14]. RPE is considered to be related to the formation and contraction of PVR membranes [15]. These complex processes are thought to comprise a series of events including the migration and proliferation of these ectopic cell sheets, the deposition of extracellular matrix, the formation of epiretinal membrane and subsequent contraction of the membrane (Fig. 1) [5, 12, 15, 16]. In recent years, accumulating evidence has shown that inflammatory mediators, such as growth factors and cytokines, in the vitreous or in the subretinal fluid, play an important role in the occurrence and development of PVR [17–25]. In this review, we focus on the role of growth factors and cytokines in the development of PVR and the current treatment for the prevention of PVR. In addition, we support that arachidonic acid metabolic cascade is important for PVR.

We searched the PubMed database using these keywords “PVR,” “inflammatory mediators,” “growth factors,” “cytokines” and “treatment.” The search was limited to studies published up to December 2019. In this report, literatures regarding inflammatory mediators in PVR and the current treatment for PVR prevention were included. Randomized controlled trials (RCTs) of medical treatments for PVR were included. Exclusion criteria included the following: literature with low relevance to this review; no detailed and comprehensive data; use of different evaluation results. According to the title and abstract, we conducted the first screening. Based on the inclusion and exclusion criteria, the second screening was performed by reading the full text of the literatures. Finally, a total of 104 articles were included in this review.

## Growth factor and cytokine hypothesis for PVR

Many laboratories reported that many growth factors and cytokines were overexpressed in the vitreous or in the subretinal fluid of PVR patients [17–25]. Based on these findings, the growth factor and cytokine hypothesis was proposed. This hypothesis proposes that abnormal expression of these inflammatory mediators drives PVR, and eventually leads to formation of the epiretinal membrane and its subsequent contraction [2, 4, 26]. These growth factors and cytokines include transforming growth factor-β (TGF-β), platelet-derived growth factor (PDGF), basic fibroblast growth factor (bFGF), vascular endothelial growth factor (VEGF), interleukin-1alpha (IL-1α), interleukin-2 (IL-2), interleukin-3 (IL-3), interleukin-6 (IL-6), tumor necrosis factor-α (TNF-α), intercellular adhesion molecule-1 (ICAM-1) and other proteins [18–23]. Among them, TGF-β, PDGF, IL-6, IL-8 and TNF-α are considered to be particularly important in this process [18–23].

### Transforming growth factor-β

The TGF-β superfamily of multifunctional mediators not only regulates cell growth and differentiation, but also promotes fibrosis and proliferation [27, 28]. Three isoforms of TGF-β, namely TGF-β1, TGF-β2 and TGF-β3, are expressed in the anterior segment of the human eye [29]. In addition, TGF-β1 was reported to be stored or activated by photoreceptors [30]. TGF-β2 is observed in the long ciliary arteries and photoreceptor outer segments [30]. In choroid and retina, TGF-β3 is expressed in isolated individual cells [30]. Hoerster et al. [31] reported that in rabbit models of PVR, TGF-β1 and TGF-β2 were dramatically up-regulated in both aqueous humor (AH) and vitreous. In addition, Kon et al. [20] observed significant elevation of TGF-β2 in human vitreous PVR samples. RPE is considered to play an essential role in the formation and contraction of PVR membranes [15]. Of note, Yao et al. [32] pointed out that PVR progression was related to TGF-β-induced epithelial–mesenchymal transition (EMT) in RPE cells. Also, Dvashi et al. [33] showed that TGF-β1 played a pivotal role in EMT of RPE via activation of transforming growth factor beta-activated kinase 1 (TAK1). Rojas et al. [34] highlighted that Smad7 was associated with PVR development and considered as a novel target for the treatment of PVR. In addition, up-regulation of Smad7, an inhibitor of TGF-β, could suppress RPE cells’ fibrogenic response to EMT [35]. Consistent with these results, pirfenidone inhibited TGF-β1-induced fibrogenesis by blocking the nuclear translocation of Smads in a human PRE cell line, ARPE-19 [36]. Later, Yang et al. [37] explored the function of long noncoding RNA (lncRNA) MALAT1 in regulating EMT in RPE cells induced by TGF-β1. Increased expression of lncRNA MALAT1 was apparently observed in RPE cells incubated with TGF-β1 [37]. However, knockdown of MALAT1 could result in the inhibition of TGF-β1-induced EMT and proliferation of RPE cells partially via the activation of Smad2/3 signaling [37]. Consequently, they believed that lncRNA MALAT1 played an important role in TGF-β1-induced EMT in human RPE cells, which might shed light on the targeted therapy of PVR [37]. Researchers explored the distribution of selected cytokine gene polymorphisms in PVR patients and attempted to identify potential genetic markers [38]. Through the detection of single-nucleotide polymorphism (SNP), they found significant difference in genotype distribution of TGF-β1 codon 10 polymorphism between PVR patients and RD patients [38]. In comparison with controls, a statistical difference in TGF-β1 codon 25 in PVR patients was observed [38]. Therefore, they considered that TGF-β1 genetic profile was associated with PVR development [38]. Furthermore, Carrington et al. [15] confirmed that TGF-β2 could stimulate RPE-mediated contraction of the retina. Additionally, neutralizing antibodies against TGF-β2 effectively inhibited RPE cell-mediated contraction [15]. Thus, TGF-β2 may represent a potential target for PVR therapies [15]. Recently, Chen and his colleagues have investigated the function of Jagged/Notch signaling in TGF-β2-mediated EMT in RPE cells [39]. They reported that knockdown of Jagged-1 expression and blockade of the Notch signaling pathway could suppress EMT in RPE cells induced by TGF-β2, which might be associated with the formation of PVR [39]. During the process of TGF-β2-mediated EMT in RPE cells, a total of 304 miRNAs were reported to have changed, of which 119 were up-regulated and 185 were down-regulated, suggesting that miRNAs might be associated with EMT in RPE cells [40]. Among them, the expression of miRNA-29b was down-regulated more than 80% [40]. Thus, Cao et al. [41] have investigated the roles of mechanical stretch and TGF-β2 in EMT in human RPE cells and the association between miRNA-29b and PVR progression. Their observations confirmed that TGF-β2 could not only induce EMT in RPE cells, but also suppress the expression of miRNA-29b in time–dose dependence [41]. Therefore, they speculated that miRNA-29b might have potential as part of a clinical strategy for PVR treatment [41]. Recently, Liu et al. [42] have investigated the effect of mouse double minute 2 (MDM2) on TGF-β2-mediated EMT in RPE cells. Importantly, their experiments demonstrated that dCas9/MDM2-sgRNA could block TGF-β2-mediated expression of MDM2 and EMT biomarkers in RPE cells [42]. In vitro, TGF-β2 contributes to EMT, collagen production and fibrosis, while decorin antagonizes TGF-β and exhibits independent anti-fibrosis properties [43]. Therefore, decorin, as an antifibrotic agent, might be a promising candidate for the inhibition of RPE fibrosis induced by TGF-β2 [43]. Mony et al. [44] explored the association between TGF-β2-mediated EMT in RPE cells and altered Na, K-ATPase expression, which was observed on the apical membrane in RPE cells. Of note, their experiments revealed that the lack of expression of Na, K-ATPase β1 subunit might be related to TGF-β2-mediated EMT and fibrosis in RPE cells [44]. After the activation of TGF-β2 signaling, EMT biomarkers were found to be induced, such as fibronectin, α-SMA pressure and actin fibers, while the decreased expression of β1 subunit was observed [44]. Interestingly, knockdown of β1 subunit in RPE cells could contribute to the mesenchymal cell morphology and induction of EMT biomarkers, indicating that the lack of Na, K-ATPase β1 subunit might be a potential trigger of TGF-β2-mediated EMT in RPE cells [44]. In addition, the reduction of Na, K-ATPase β1 mRNA was negatively correlated with the level of HIF-1α [44]. It has been reported that the binding of HIF-1α with Na, K-ATPase β1 promoter and the inhibition of HIF-1α activity could block the decrease of Na, K-ATPase β1 mediated by TGF-β2, suggesting that HIF-1α might participate in the regulation of Na, K-ATPase β1 during the EMT in RPE cells [44]. Due to the abnormal expression of TGF-β in PVR, and the efficacy of TGF-β inhibitors to prevent fibrogenic response, we agree that TGF-β is likely to be associated with the development of PVR and propose TGF-β as a candidate target for PVR therapies. However, this hypothesis still needs to be validated in a more precise system, such as an animal model of PVR in which TGF-β is knocked out or knocked down.

### Platelet-derived growth factor (PDGF)

Like TGF-β, many laboratories have demonstrated that PDGF and PDGF receptor (PDGFR) might participate in PVR development. Cui et al. [45] and Robbins et al. [21] confirmed that levels of both PDGF and PDGFR were significantly higher in PVR membranes than in control membranes. Both PDGF and PDGFR could be secreted by RPE and glial cells [21, 45]. In addition, subsequent studies indicated that the level of PDGF was dramatically elevated in the vitreous of PVR patients [46]. Moreover, animal experiments supported the hypothesis that PDGF and PDGFR might participate in the complex process of PVR development [46, 47]. Most rabbit models of PVR are established by injection of fibroblasts into the vitreous [48]. In these animals, PDGF is undetectable in the vitreous of the control group, while overexpression of PDGF, especially PDGF-C, is observed in the vitreous of model rabbits [46]. Coincidentally, vitreous is reported to contain the protease necessary for PDGF-C activation [47]. Furthermore, inhibition of PDGFR expression could reduce cellular PVR formation [49, 50]. Among three isoforms of PDGFR, namely PDGFRα, PDGFRβ and PDGFRαβ, PDGF-C can activate PDGFRα and PDGFRαβ [46, 51]. Previous findings indicated that PDGFRα could be capable of triggering the events that ultimately lead to experimental PVR [52]. Recently, Zhang et al. [53] have demonstrated that crocetin could act as an effective inhibitor of PDGF-BB-induced proliferation and migration of RPE. Taken together, PDGF-C and its receptor PDGFRα are considered more important in the formation of PVR. Actually, besides PDGF, non-PDGF factors such as epidermal growth factor (EGF), basic fibroblast growth factor (bFGF), hepatocyte growth factor (HGF) and insulin could be capable of activating PDGFR indirectly [2, 4]. Direct activation of PDGFRα is driven by PDGF, causing subsequent activation of receptor’s intrinsic kinase [4]. Non-PDGFs could activate PDGFRα through reactive oxygen species (ROS) and Src family kinases (SFKs) [2, 4]. Non-PDGFs could engage its receptors and increase the level of ROS, resulting in the activation of SFK and phosphorylation of PDGFRα [2, 4]. Indirect activation of PDGFRα pathway by Non-PDGFs could prolong the activity of signaling enzymes such as PI3K/Akt, inhibit the expression of p53 gene and thereby promote cell survival and proliferation [2, 4]. In recent years, ultrasound-targeted microbubble destruction (UTMD) and RNA interference (RNAi) have shed light on gene therapy of eye diseases and become a new research hotspot [54]. Interestingly, studies have shown that the combination of UTMD and recombinant adeno-associated virus (rAAV)-mediated RNAi targeting TGFβ2 and PDGF-B could represent a novel approach to inhibit PVR proliferation [54]. As mentioned above, both clinical studies and animal experiments support the theory that PDGF and PDGFR are implicated in the occurrence and development of PVR. These studies highlight more therapeutic targets for PVR prevention. However, a targeted therapy has not yet reached clinical trials.

### Interleukins

Interleukin can be secreted by a variety of cells and stimulate cellular responses. In addition to activating and regulating immune cells, interleukin is a critical signal for abnormal proliferation, differentiation of T- and B-lymphocytes and enhancement of inflammation [18, 55, 56]. In recent years, interleukin has been associated with the occurrence and development of PVR [18, 19, 22]. As a marker of acute inflammatory reaction, IL-6 exhibits a wide range of biologic activities. Not only can IL-6 attract chemokines, it can also recruit leukocytes to local inflammatory sites [18, 57]. Using a multiplex bead-based immunoassay, Ricker et al. [18] investigated interleukin levels in the subretinal fluid of 21 PVR patients and 54 RRD patients. A significant increase in IL-6 was observed in the PVR cases when compared with controls [18]. Consistent with these data, Symeonidis et al. [19] reported that IL-6 was up-regulated in both vitreous and subretinal fluid in the PVR group. Most importantly, they demonstrated that IL-6 levels were positively correlated with the extent and duration of RRD and PVR grade [19]. Previous findings have shown that RPE cells, forming part of the blood–retinal barrier, were potent producers of various inflammatory cytokines including IL-6 [58]. Also, IL-6 could be secreted by other cell types. Similarly, Kon et al. [20] and EI-Ghrably et al. [59] demonstrated that IL-6 was a critical mediator in the complex process of postoperative PVR development. The function of IL-6 signal transduction depends on the gp130 family [60]. Recently, Lumi et al. [61] have assessed the differences in genotype distributions of single-nucleotide polymorphisms (SNPs) between PVR cases and RRD cases. Interestingly, the significant difference in genotype distribution of rs1800795 (IL-6) could be observed between PVR cases and controls [61]. Furthermore, in IL-6 (rs1800795), RRD patients with the G allele possibly had a higher risk of progressing into PVR than patients with the C gene [61]. Unfortunately, after adjustment by multiple hypothesis testing, genotype distribution of IL-6 (rs1800795) had no statistical difference between two groups [61]. Herein, in order to clarify the genetic contribution to PVR, further investigations are needed. In addition to the recruitment and activation of neutrophils, IL-8 plays a pivotal role in the regulation of inflammatory responses and mediation of angiogenesis. Interestingly, the IL-8 levels were elevated in PVR cases [62, 63]. However, IL-8 levels did not correlated with PVR grade [64]. Later, Rasier et al. [22] measured the levels of IL-8 in 22 RRD patients and 12 cases of epiretinal membranes and macular holes. Similarly, IL-8 levels were significantly elevated in the RRD group [22]. Thus, Rasier et al. [22] speculated that IL-8 might contribute to the development of PVR. Previous report has indicated that the mRNA encoding IL-8 was obviously observed in subretinal fluid and vitreous in PVR patients [65]. Perhaps in the future, neutralizing the expression of cytokines may inhibit development of PVR. Of course, this requires further exploration.

### Tumor necrosis factor alpha (TNF-α)

TNF-α, a member of the TNF ligand superfamily, has a wide range of biologic activities and is implicated in the regulation of inflammatory response, apoptosis and cellular proliferation [66–68]. Limb et al. [23] investigated the distribution of TNF-α within epiretinal membranes in 26 PVR patients. They reported that approximately 85% of the 26 epiretinal membranes contained TNF-α not intracellularly and in the extracellular matrix [23]. Herein, they maintained that cytokine-mediated inflammatory pathways were critical for the development of PVR [23]. In line with this result, the vitreous levels of soluble TNF-receptors (sTNF-Rs, sTNF-RI, sTNF-RII) and TNF-α were found to be higher in 30 PVR cases than in 30 RRD cases [69]. Importantly, levels of sTNF-Rs were correlated with the duration of retinal detachment (RD) and grade of severity. Therefore, they hypothesized that sTNF-Rs-mediated regulation of TNF-α activity might effectively limit severity of retinal proliferation [69]. Previous studies have shown that adhesion and migration of RPE cells to provisional extracellular matrices(ECM) played a critical role in the formation of epiretinal membrane in PVR [70]. Jin and his colleagues investigated the effects of TNF-α on adhesion and migration of RPE cells to ECM and explored the mechanism of this response [70]. They pointed out that TNF-α could regulate RPE cells to ECM through the activation of MAPK pathway [70]. Therefore, they believed that TNF-α and MAPK might be the potential target for PVR therapy [70]. Recently, Wang et al. [71] have reported that it was through the activation of Akt/mTORC1 signaling that TNF-α could promote the migration of RPE cells by inducing the expression of matrix metallopeptidase (MMP)-9. Moreover, the expression of TNF-α mRNA-positive cells was obtained in epiretinal membranes in PVR patients, which might provide new direction for the treatment of PVR [72]. Although the association between various SNPs in TNF, such as rs1800629 (TNF), and the increased risk of PVR was reported, previous results could not be replicated [61]. Based on these results, we believe that TNF-α is implicated in the development of PVR and thus represents a therapeutic target. It will be interesting to investigate whether TNF-a-specific inhibitors, such as infliximab (remicade), could prevent or reduce PVR.

#### Other mediators: arachidonic acid metabolite

Besides the direct regulation of growth factors, cell functions could also be mediated through indirect mechanism, such as those regulated by arachidonic acid metabolites [73]. Observations have provided a close link between arachidonic acid metabolites and intraocular inflammation [73]. In vivo, through the cyclooxygenase (COX) pathway, lipoxygenase enzymes pathway and cytochrome P450 pathway, arachidonic acid could be metabolized to prostaglandins, thromboxane, leukotriene, epoxyeicosatrienoic acids and so on [73, 74]. In addition to regulating intraocular pressure, prostaglandins are associated with intraocular inflammation and the breakdown of the blood–retinal barrier [75]. Kahler et al. [73] have shown the release of arachidonic acid metabolites was found to increase rapidly and continuously after exposure of human fibroblasts to vitreous from PVR patients. Also, their experiments revealed that prostaglandin E_2_ (PGE_2_), which could cause continuous breakdown of aqueous blood–retinal barrier, was significantly increased in PVR cases [73]. However, as compared with controls, the release of prostaglandin I_2_, leukotriene B_4_ and thromboxane A_2_ was not significantly affected [73]. Also, their study demonstrated that COX inhibitors, such as acetylsalicylic acid, could effectively inhibit the release of arachidonic acid metabolites, which might provide a novel therapeutic strategy for intraocular fibrosis [73]. In addition, the exposure of RPE cells to vitreous could lead to elevated expression of membrane-associated prostaglandin E-synthase (mPGES) and cyclooxygenase (COX)-2, both of which were considered as the critical enzymes in the synthesis of prostaglandin E_2_ from arachidonic acid [75]. Also, Parapuram et al. [75] pointed out that the inhibition of PGE_2_ production by suppressing the synthesis of mPGES could lead to the reduction in inflammation, which might provide a potential therapeutic approach to prevent the progress of PVR. More importantly, they speculated that the effect of the vitreous on the prostaglandin pathway might be related to growth factors [75]. In RPE cells, IL-1β was associated with the up-regulation of COX-2 and PGE_2_ expression [75]. The combined application of IL-1β and TNF-α or IFN-r could result in higher expression of PGE_2_ than treatment with IL-1β alone [75]. Recently, scholars have identified that the levels of prostaglandin D_2_ synthase (PGD_2_S) were obviously increased in human PVR proteome analysis and were highly elevated in experimental PVR retina [76]. PGD_2_S, known as the key enzyme in the synthesis of prostaglandin D_2_ (PGD_2_), is connected with inflammatory response and retinal apoptosis [76]. It is known that COXs can synthesize prostaglandins from arachidonic acid [77]. On the other hand, prostaglandins can also lead to the increased synthesis of COX-2 by positive feedback mechanism [78]. Previous studies have confirmed that the expression COX-1 and COX-2 was detectable in the proliferative membranes of PVR patients [79]. At the early stages of PVR development, the administration of lornoxicam, known as nonsteroidal anti-inflammatory drugs (NSAIDs), could not only inhibit the expression of COXs in the retina and choroid, but also reduce the frequency of membrane formation by 31–43% in PVR models [77]. Recent reports have shown that calcium-independent phospholipase A_2_ could regulate the proliferation of RPE cells and be identified in most transformed PRE cells in the membranes of PVR patients [80]. Thus, these observations have indicated that calcium-independent phospholipase A_2_ was associated with the formation of PVR membranes, which might shed light on the treatment of PVR [80]. It is well known that steroid drugs can block the effect of phospholipase A_2_ and inhibit the metabolism of arachidonic acid cascade [77].

## Treatment

### Surgery

Currently, surgery is the standard treatment for PVR [9]. The aim of PVR surgery is to reattach the retina and achieve satisfactory visual acuity. Although tremendous advances in vitreoretinal surgical techniques, such as 27-G transconjunctival sutureless vitrectomy system and noncontact wide-angle viewing system [57, 81], have been made over recent decades, both anatomic success and functional success rates are still unsatisfactory. In prospective studies, postoperative PVR occurs in 4% to 34% of cases [82]. Herein, novel therapeutic strategies are required to prevent or halt the progression of PVR.

### Pharmacological adjuvant therapies

Several pharmacological adjuvant therapies have been applied to improve surgical outcomes, including anti-inflammatory, anti-proliferative, antineoplastic, anti-growth factor agents and statins. (Table 1) The clinical trials (RCTs) for the evaluation of pharmacological adjuvant therapies of PVR are summarized in Table 2.

**Table 1:** Pharmacological adjuvant therapies

Targets	Adjuvant agents
Anti-inflammatory agents	Steroid (triamcinolone acetonide, crystalline cortisone, slow-release dexamethasone)
Anti-proliferative/antineoplastic agents	5-Fluorouracil, daunorubicin, taxol, colchicine, retinoic acid, ribozymes
Anti-growth factor pathway inhibitors	Decorin, fasudil, AG1295
Statins	Simvastatin

Pharmacological adjuvant therapies include anti-inflammatory agents, anti-proliferative/antineoplastic agents, anti-growth factor pathway inhibitors and statins. Various types of adjuvant agents are listed above

**Table 2:** Clinical trials (RCTs) for the evaluation of pharmacological adjuvant therapies of PVR

References	Agents	Dosage	Patients	Follow-up interval	Results
Ahmadieh et al. [86]	TA	Intravitreal injection of 4 mg TA at the end of PPV	75 patients with PVR Grade C	6 months	No significant difference in retina reattachment and postoperative visual acuity between groups
Yamakiri et al. [87]	TA	TA-assisted PPV (40 mg/5 mL solution)	774 patients	1 year	No significant difference in vision, postoperative complications and adverse events between groups
Banerjee et al. [88]	Slow-release dexamethasone	Injection of 0.7 mg slow-release dexamethasone during PPV and silicone oil removal	140 patients with PVR Grade C	2 years	Similar results in anatomic success but greater reduction in cystoid macular edema at 6 months
Asaria et al. [90]	5-FU and LMWH	200 μg/ml 5-FU 5 IU/ml LMWH	174 patients	6 months	A significant reduction in incidence of postoperative PVR and reoperation rate in the treatment group
Wickham et al. [91]	5-FU and LMWH	200 μg/ml 5-FU 5 IU/ml LMWH	641 RRD patients	6 months	No improvement in the anatomic or visual success rate between groups; worse visual acuity in patients with macula off detachment in the treatment group
Wiedemann et al. [95]	Daunorubicin	10 min infusion of 7.5 μg/ml daunorubicin in balance solution	286 patients with PVR Grade C	1 year	No significant difference in anatomic success rate between groups; fewer reoperations in the treatment group
Kumar et al. [96]	Daunorubicin	Intravitreal injection of 5 μg daunorubicin	30 patients with PVR Grade D	3 months	Increased reattachment rate and better visual acuity in the treatment group

5-FU 5-fluorouracil; LMWH low molecular weight heparin; PVR proliferative vitreoretinopathy; RRD rhegmatogenous retinal detachment; TA triamcinolone acetonide

#### Anti-inflammatory agents

In order to inhibit inflammation in PVR, more attention has been paid to anti-inflammatory agents, especially steroids. Previously, Hui et al. [83] evaluated the efficacy of triamcinolone acetonide (TA) in the prevention of PVR in rabbit models of PVR. The incidence of RD decreased from 77.5% to 13.3% following intravitreal injection of 1 mg TA [83]. Later, Rubsamen et al. [84] demonstrated a 73% reduction in the RD rate following periocular steroid injection in animal models of PVR. In spite of the great success achieved with steroids in animal experiments, no clinical breakthroughs have yet been made. Recently, intravitreal injection of crystalline cortisone has been found to effectively reduce postoperative intraocular inflammation and does not damage the intraocular structure in PVR patients [85]. In a prospective randomized controlled trial, 75 eyes with RRD and PVR grade C were randomly divided into TA treatment group and control group [86]. After 6 months, no significant difference in the retina reattachment rate and postoperative visual acuity between two groups was achieved [86]. Similarly, Yamakiri et al. [87] evaluated the one-year results of using TA in PPV in another multicenter prospective controlled clinical trial. This study population consisted of 391 eyes that underwent TA-assisted PPV and 383 control eyes that underwent PPV [87]. Unfortunately, after one year, there was no statistical difference in vision, postoperative complications and adverse events between two groups [87]. Recently, Banerjee et al. [88] have explored the effect of adjunctive slow-release dexamethasone implant on PVR cases. A total of 140 PVR eyes that underwent PPV were randomized into standard group and treatment group [88]. An adjunctive slow-release dexamethasone implant in PVR cases, during PPV and silicone oil removal, could reduce cystoid macular edema but not improve the anatomic success rate [88].

#### Anti-proliferative/antineoplastic agents

PVR is described as a complex process occurring secondary to RRD and following the proliferation of ectopic cell sheets and the formation of epiretinal membrane. Thus, attempts to prevent proliferation, antineoplastic agents, like 5-fluorouracil (5-FU), daunorubicin, taxol, colchicine, retinoic acid, ribozymes and so on, have been widely studied. Among all these antineoplastic agents, 5-FU and daunorubicin are most widely used. Although 5-FU significantly prevented PVR in animal experiments, recent two randomized controlled trials were inconsistent and controversial [89–91]. Asaria et al. [90] demonstrated a significant reduction in incidence of postoperative PVR and reoperation rate in the 5-FU and low molecular weight heparin (LMWH) therapy group. However, Wickham et al. [91] noted that there was no statistical difference in anatomic or visual success rates between 5-FU and LMWH therapy group and controls. After adjuvant medication, worse final visual acuity was observed in patients with macula off detachment [91]. In addition, a Cochrane’s review points out a lack of sufficient evidence of the capacity of 5-FU and LMWH to halt progression of PVR [92]. In experimental models, daunorubicin is considered to be an effective pharmacologic inhibitor of PVR progression [93, 94]. However, in recent human trails, their conclusions remained inconsistent and controversial [95, 96]. Wiedemann et al. [95] pointed out that no significant difference in anatomic success rate was obtained between groups after 6 months. Fewer reoperations were observed in daunorubicin group after one year [95]. Kumar et al. [96] indicated that the increased reattachment rate and better visual acuity were found in PVR patients treated with daunorubicin at 3-month follow-up. Thus, additional clinical trials will be necessary to evaluate the efficacy and safety of daunorubicin as an adjunctive treatment.

#### Anti-growth factor pathway inhibitors

Based on the growth factor and cytokine hypothesis, inhibitors with anti-growth factor pathway activity have been considered promising candidates for PVR treatment. Decorin, a naturally occurring TGF-β inhibitor, has shown positive results in terms of reducing fibrosis and tractional retinal detachment (TRD) development in rabbit models of PVR [97]. Thus, adjuvant decorin application during PPV may represent an effective therapeutic approach in prevention of PVR reactions without apparent histopathological toxicity to the eyes [97]. A key downstream mediator of TGF-β, Rho-kinase (ROCK) (which is implicated in cell adhesion, migration, proliferation and apoptosis [98]) is also a target for PVR therapy. Indeed, fasudil, a potent and selective ROCK inhibitor, has already proved to be effective in halting PVR progression in rabbit models [98]. Herein, ROCK inhibition provides a novel therapeutic strategy for the management of PVR and more clinical trials with fasudil need to be performed. AG1295, a specific inhibitor of PDGFR, has shown promising results in animal experiments [50]. In a recent study, Zheng et al. [50] used rabbit models of PVR to investigate the effect of AG1295 on PVR development. Amazingly, development of TRD was markedly attenuated in the AG1295-treated group [50]. Therefore, AG1295 appeared to be an efficacious therapeutic target for preventing PVR progression [50].

#### Statins

Statins are widely prescribed to reduce the risk of cardiovascular events [99]. Administration of statins may also ameliorate osteoporosis and the growth and metastasis of cancer [100, 101]. Recently, the application of statins in vitreoretinal diseases, such as PDR and RVR, has become a research hot spot [102–104]. In the last decade, statins have been reported to effectively prevent proliferation of PVR and reduce the need for further surgical interventions in postoperative RRD patients [103, 104]. Therefore, statins may represent a new strategy to prevent PVR development. Importantly, Kawahara et al. [102] demonstrated that vitreous samples from PVR and PDR patients enhanced the phosphorylation of myosin light chain and gel contraction, while simvastatin could almost completely inhibit these phenomena in a dose-dependent manner without apparent toxicity. In addition, simvastatin could suppress the development of PVR in animal models [102]. The PVR models were established by injection of fibroblasts into each rabbit’s right eye. Six rabbits in the first group received intravitreal injection of 0.1 mL equilibrium salt solution, and seven rabbits in the second and third groups received intravitreal injection of 0.1 mL vehicle containing 5 μmol/l and 15 μmol/l of simvastatin, respectively [102]. As a result, the first group developed PVR, forming proliferative membranes, while simvastatin inhibited development of PVR [102]. In a retrospective study, researchers compared the levels of inflammatory mediators, including angiopoietin (ANGPT)-1 and -2, TGF-β1, VEGF and matrix metalloproteinase (MMP)-2 and (MMP)-9, between the vitreous of 14 RRD patients treated with statins and 82 RRD patients without statin treatment [103]. Amazingly, they found that the levels of ANGPT-2, VEGF and MMP-2 were significantly lower in the eyes of RRD patients taking statins [103]. Moreover, they noted that RRD patients taking statins tended to have better one-month best-corrected visual acuity (BCVA) [103]. The implications of these findings are limited by the study’s sample size. Therefore, large RTCs are urgently needed to evaluate the efficacy and safety of statins in the treatment of PVR. Later, Loukovaara et al. [104] analyzed the relationship between statins and further surgical intervention after primary vitrectomy in 5709 patients with vitreoretinal disease. Among them, there were 1916 RRD patients, accounting for nearly 1/3 of the total patients [104]. In this study, preoperative use of statins was associated with a 28% reduction in the rate of further surgical intervention in RRD patients [104]. Thus, they suggest that statins may represent an efficacious treatment for PVR [104]. However, questions remain about use of statins. The role of statin therapy in PVR is not yet fully understood. Which age groups should take statins, for how long and at what dose? Whether preoperative oral statins or intraoperative intravitreal injection of statins can obtain higher surgical success rates and lower the rate of further surgical intervention remains to be seen. Thus, more animal experiments and RTCs are necessary to explore the use of statins as adjuvant drugs in the treatment of PVR.

## Conclusion

Despite great advances in surgical techniques, PVR remains a common cause of severe vision loss. This sight-threatening disorder comprises a series of events including cell migration, cell proliferation and formation of epiretinal membrane [5, 12, 15]. With improved understanding of the pathogenesis of PVR, many laboratories have proposed growth factor and cytokine hypothesis and presented sufficient evidences. These growth factors and cytokines act as signaling molecules to trigger more mediator secretion, amplify the inflammatory reaction and eventually lead to the formation of proliferative membranes. More importantly, the identification of inflammatory mediators provides novel and efficacious therapeutic targets for the treatment of PVR. Various pharmacological adjuvant agents have been extensively investigated. Some agents have been found to ameliorate PVR progression in animal models, but the limited clinical studies were not conclusive—perhaps due to small sample size or short-term follow-up time. Certainly, more work needs to be done to halt the progression of PVR, improve the rate of retinal reattachment and achieve satisfactory vision.

## Abbreviations

AH: Aqueous humor
ANGPT: Angiopoietin
BCVA: Best-corrected visual acuity
COX: Cyclooxygenase
ECM: Extracellular matrices
EMT: Epithelial–mesenchymal transition
EGF: Epidermal growth factor
bFGF: Basic fibroblast growth factor
5-FU: 5-Fluorouracil
HGF: Hepatocyte growth factor
ICAM-1: Intercellular adhesion molecule-1
IL: Interleukin
LMWH: Low molecular weight heparin
MDM2: Mouse double minute 2
MMP: Matrix metalloproteinase
NSAIDs: Nonsteroidal anti-inflammatory drugs
PDGF: Platelet-derived growth factor
PDGFR: PDGF receptor
PGD_2_: Prostaglandin D_2_
PGD_2_S: Prostaglandin D_2_ synthase
PGE_2_: Prostaglandin E_2_
mPGES: Membrane-associated prostaglandin E-synthase
PPV: Pars plana vitrectomy
PVR: Proliferative vitreoretinopathy
RCTs: Randomized clinical trials
RD: Retinal detachment
lncRNA: Long noncoding RNA
RNAi: RNA interference
ROCK: Rho-kinase
ROS: Reactive oxygen species
RPE: Retinal pigment epithelial
RRD: Rhegmatogenous retinal detachment
rAAV: Recombinant adeno-associated virus
SKFs: Src family kinases
SNP: Single-nucleotide polymorphism
sTNF-R: Soluble TNF-receptor
TA: Triamcinolone acetonide
TAK1: Transforming growth factor beta-activated kinase 1
TGF-β: Transforming growth factor-β
TNF-α: Tumor necrosis factor-α
TRD: Tractional retinal detachment
UTMD: Ultrasound-targeted microbubble destruction
VEGF: Vascular endothelial growth factor
